# Dysplasia of the tricuspid valve leading to recurrent atrial flutter and fibrillation: a case report

**DOI:** 10.1093/ehjcr/ytae675

**Published:** 2024-12-20

**Authors:** Taemi Yoshida, Edmund Gatterer, Andreas Strouhal, Marieluise Harrer, Claudia Stöllberger

**Affiliations:** Department of Cardiology, Klinik Landstrasse, Juchgasse 25, A-1030 Wien, Austria; Department of Cardiology, Klinik Landstrasse, Juchgasse 25, A-1030 Wien, Austria; Department of Cardiology, Department of Cardiac and Vascular Surgery, Klinik Floridsdorf, Brünnerstrasse 68, A-1210 Wien, Austria; Department of Cardiology, Department of Cardiac and Vascular Surgery, Klinik Floridsdorf, Brünnerstrasse 68, A-1210 Wien, Austria; Department of Cardiology, Klinik Landstrasse, Juchgasse 25, A-1030 Wien, Austria

**Keywords:** Atrial flutter, Cavotricuspid isthmus ablation, Tricuspid valve regurgitation, 3D-echocardiography, Atrial fibrillation

## Abstract

**Background:**

Atrial flutter (AFL) is usually effectively treated by cavotricuspid isthmus (CTI) ablation. If AFL recurs despite ablation, there is risk of progression to atrial fibrillation (AF) and clinicians should consider underlying structural heart diseases. This consideration becomes especially critical when right-heart-chambers are dilated.

**Case summary:**

A 50-year-old man presented with palpitations due to AFL. Fifteen years earlier, after polytrauma, mild tricuspid regurgitation (TR) and pericardial effusion had been diagnosed on transthoracic echocardiography (TTE). At present, TTE showed dilated right-heart-chambers and moderate TR. Despite two CTI-ablations, he developed AF for which he underwent pulmonary vein isolation (PVI). A further ablation was performed because of right-sided AFL due to transcrista conduction. Atrial fibrillation recurred, accompanied by heart failure. Tricuspid regurgitation severity and right-heart-chamber dilatation worsened. Finally, 3D-transoesophageal echocardiography (3D-TEE), performed 20 years after the first TTE, revealed that TR was due to restriction of the septal leaflet. The patient underwent surgery. The tricuspid valve was repaired by ring annuloplasty and a cleft between the anterior and septal leaflets was closed. Three years post-operatively, he is asymptomatic with chronic AF but no recurrent AFL. Transthoracic echocardiography shows only mild TR, though the right-heart-chambers remain dilated, likely due to long-standing TR.

**Discussion:**

Tricuspid regurgitation and AFL/AF have a bidirectional relationship. Tricuspid regurgitation can both cause and result from AFL/AF. Structural heart diseases, including post-traumatic valve damage, should be considered in patients with recurrent AFL despite CTI-ablation and progression to AF. In cases with TR and right-heart-chamber enlargement, 3D-TEE is essential for accurate diagnosis and should be performed without delay.

**Learning points**

- If atrial flutter recurs despite successful ablations and progress to atrial fibrillation, a structural heart disease should be suspected.
- For morphological inspection of the tricuspid valve, 3D-transoesophageal echocardiography should be considered.

## Introduction

Atrial flutter (AFL) can be treated effectively with cavotricuspid isthmus (CTI) ablation.^1^ However, the role of valvular disorders, particularly those involving the tricuspid valve, in the development of AFL or atrial fibrillation (AF), is only marginally mentioned in the guidelines of the European Society of Cardiology (ESC).^1,2^ We present a case of recurrent AFL, despite two CTI-ablations and ablation at the crista terminalis, which progressed to AF and recurred even after pulmonary vein isolation (PVI), due to a structural defect of the tricuspid valve. This defect was only identified using 3D-transoesophageal echocardiography (3D-TEE).^3^

## Summary figure

**Figure:**
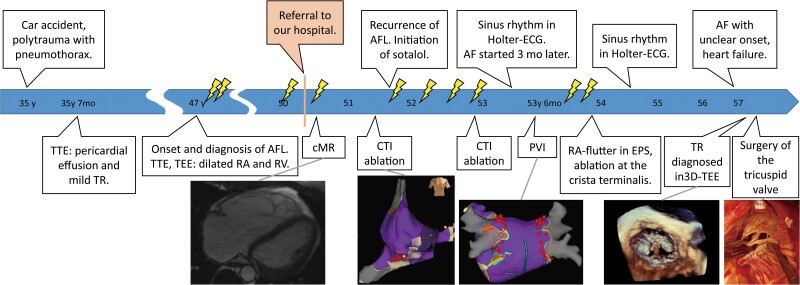


## Case presentation

**Figure 1:**
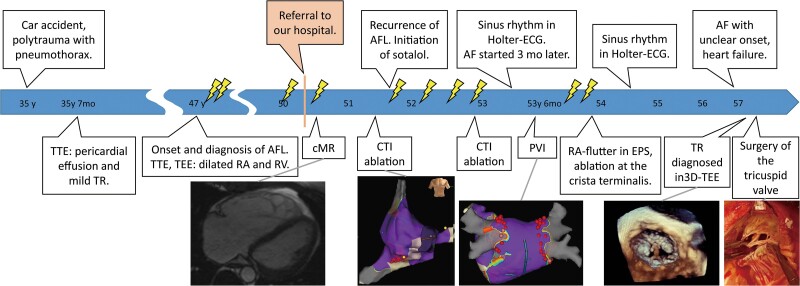
Summary of the time course over 22 years, starting with a car accident at the age of 35 years. Lightning, electrical or medical cardioversion; y, age in years; mo, months; AFL, atrial flutter; AF, atrial fibrillation; cMR, cardiac magnetic resonance tomography; CTI, cavotricuspid isthmus; PVI, pulmonary vein isolation; ECG, electrocardiogram; EPS, electrophysiological study; TEE, transoesophageal echocardiography; TTE, transthoracic echocardiography; 3D-TEE, three-dimensional transoesophageal echocardiography; RA, right atrium; RV, right ventricle; TR, tricuspid regurgitation.

The patient is a 50-year-old Caucasian man with a 3-year history of hypertension, referred to our hospital for episodes of AFL, accompanied by palpitations (*Figure 1*). Fifteen years earlier, the patient had suffered polytrauma, including a left-sided pneumothorax and pericarditis. At that time, transthoracic echocardiography (TTE) revealed mild tricuspid regurgitation (TR) and 13 mm pericardial effusion. Twelve years later, the patient began experiencing palpitations, which could last several days, prompting visits to emergency departments for electrical or pharmacological cardioversions. These palpitations impaired the patient’s quality of life and affected his work as a farmer. Atrial flutter was diagnosed, and the patient underwent three electrical cardioversions. Transthoracic echocardiography showed an enlarged right atrium (RA) and right ventricle (RV) with moderate TR, but no signs of pressure overload or pulmonary hypertension. An atrial septal defect (ASD) was ruled out by TEE using a multiplane probe.

**Figure 2:**
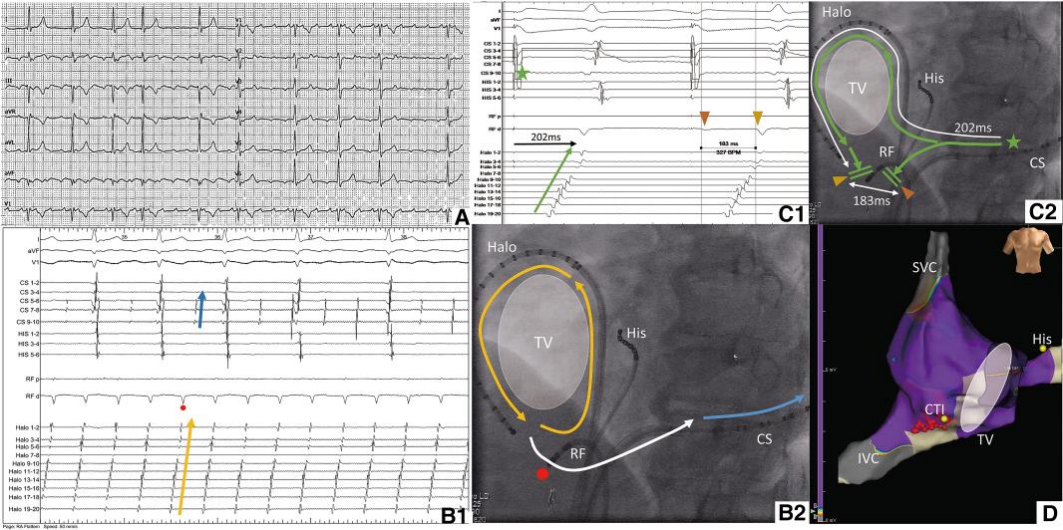
Electrocardiograms and electrophysiologic findings, registered during the first ablation. (*A*) The 12-lead electrocardiogram shows atrial flutter with a ventricular frequency of 70 bpm, normal axis, incomplete right bundle branch block, and normal repolarization. The atrial activation shows a sawtooth pattern with a cycle length of 270 ms, negative in inferior leads, with a slow downward slope followed by a fast upward slope, and a positive F-wave in V_1_. (*B1*) Intracardiac electrocardiogram of atrial flutter induced during cavotricuspid isthmus (CTI) ablation. The activation, registered by the halo catheter, is depicted as a yellow arrow and runs from the proximal to the distal part. The activation registered by the coronary sinus (CS)-catheter is depicted by the blue arrow and runs from the proximal to the distal part. The signals at the coronary sinus-catheter are partly not registered because of poor contact due to the dilatation of the right atrium and the coronary sinus. The ablation catheter (red point) is placed in the cavotricuspid isthmus. HIS, his bundle; RF, ablation catheter; Halo, 20-pole halo catheter positioned in the right atrium along the tricuspid ring. (*B2*) Fluoroscopic picture in LAO (left anterior oblique) 50° projection with schematic drawing of the activation, as recorded in (*B1*). The yellow arrow shows the activation running counterclockwise around the tricuspid valve (TV). The broken yellow arrow is the assumed activation around the tricuspid valve. The blue arrow shows the activation running from the proximal part to the distal part of the coronary sinus-catheter. The red point shows the tip of the RF catheter placed on the cavotricuspid isthmus. The white broken line indicates the assumed activation passing the cavotricuspid isthmus. (*C1*) Intracardiac ECG recorded at the end of the first cavotricuspid isthmus ablation during pacing from the proximal pole of the coronary sinus-catheter. The green star indicates the point of pacing. The activation on the Halo catheter runs from the proximal part to distal with an activation time from coronary sinus to cavotricuspid isthmus measured 202 ms. Double potentials (the orange and the yellow triangles) are also recorded on the RF-catheter with the time between the two signals measured 183 ms. (*C2*) Fluoroscopic picture in LAO 50° projection with schematic drawing of the activation, as recorded in (*C1*). The green star shows the site of pacing. The long activation time of 202 ms from the proximal coronary sinus to distal Halo reflects that the cavotricuspid isthmus line (where the RF catheter is positioned) is blocked. The first farfield-signal recorded on the RF-catheter (orange triangle) indicates early activation septal from the cavotricuspid isthmus line and the second farfield-signal (yellow triangle) lateral from the cavotricuspid isthmus line. The long time (183 ms) between the two signals is a proof, that the cavotricuspid isthmus line is electrically blocked. (*D*) Voltage map of the right atrium in anterior–posterior projection. The purple colour indicates a voltage >0.5 mV, which is considered to be normal. The red points are the ablation points. His indicates the place where the His-potential was recorded. SVC, superior vena cava; IVC, inferior vena cava; CTI, cavotricuspid isthmus; TV, tricuspid valve.

**Figure 3:**
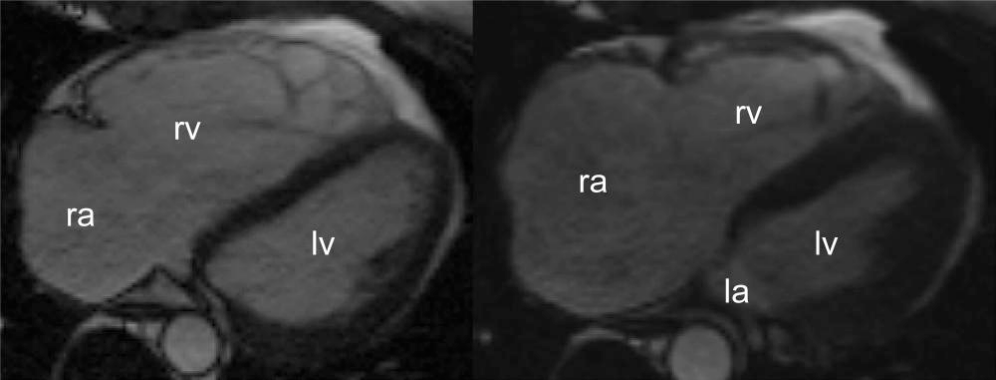
Cardiac magnetic resonance tomography in the end-diastolic phase (left) and in the end-systolic phase (right) shows significant dilatation of both the right atrium and right ventricle. Cine imaging is available in the supplementary material online. ra, right atrium; rv, right ventricle; la, left atrium; lv, left ventricle.

The patient was treated with phenprocoumon and metoprolol 95 mg/day. Physical examination revealed splitting of the second heart sound and a holosystolic murmur in the right fourth intercostal space. The electrocardiogram (ECG) showed AFL with a ventricular rate of 70 bpm, normal axis, incomplete right bundle branch block, and normal repolarization. Atrial activation displayed a sawtooth pattern with a cycle length of 270 ms, negative in the inferior leads, with a slow downward slope followed by a fast upward slope, and a positive F-wave in V_1_. These features indicated typical AFL with counterclockwise activation around the tricuspid valve (*Figure 2*). Cardiac magnetic resonance imaging (cMR) showed dilated RA and RV, with an end-diastolic volume of 465 mL, a right ventricular ejection fraction of 49% (normal: >47%) and signs of volume overload (*Figure 3*). The tricuspid valve was not well visualized, and there were no aneurysms or focal wall motion abnormalities.

**Figure 4:**
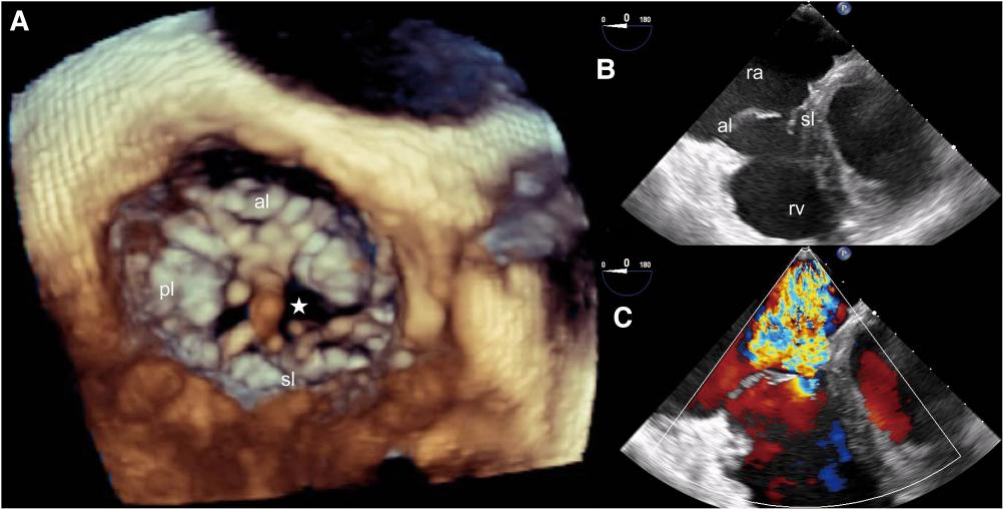
Tricuspid valve in the 3D-transoesophageal echocardiography (*A*) and in the 2D-transoesophageal echocardiography (*B* and *C* with colour Doppler) in the end-diastolic phase shows restriction of the septal leaflet, the regurgitant orifice (star), and the jet. Video clips are available in the supplementary material online. al, anterior leaflet; sl, septal leaflet; pl, posterior leaflet; ra, right atrium; rv, right ventricle.

Given the suspected typical AFL, CTI-ablation was performed (*Figure 2*). One month after the initially successful ablation, AFL recurred. Metoprolol was switched to sotalol 160 mg/day, but symptoms persisted. In the electrophysiological study (EPS), reconduction of the CTI was detected, necessitating another ablation. Six months later, the patient experienced palpitations and dyspnoea due to AF. Pulmonary vein isolation using radiofrequency was performed, but atypical AFL recurred after 8 months. As the patient remained symptomatic with AFL, several cardioversions were conducted, but the patient only maintained sinus rhythm for a short period. In a further EPS, right AFL due to a gap in the crista terminalis was diagnosed,^4^ and an ablation was performed at the caudal part of the crista terminalis, where fractionated signals were found. After 6 months, the patient was in sinus rhythm on sotalol. However, 18 months later, the patient returned with peripheral oedema and worsening dyspnoea. The ECG showed AF of unknown onset. Transthoracic echocardiography indicated an increase in the severity of TR, prompting referral to another hospital for 3D-TEE to investigate the aetiology of TR. 3D-TEE revealed restriction of the septal tricuspid leaflet due to short chordae tendineae pulling the leaflet toward the interventricular septum (*Figure 4*). The regurgitation was worsened by ring dilatation, leading to impaired leaflet coaptation, also known as ‘pseudo-prolapse’.^5^ Based on these findings and the worsening right-heart-failure with liver vein congestion, the patient was referred for surgery.

**Figure 5:**
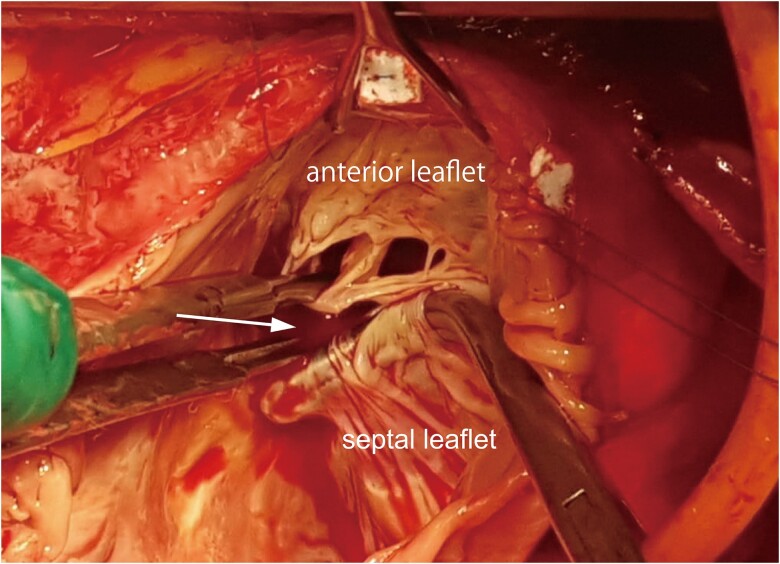
Intraoperative picture of the tricuspid valve seen from the right atrium. The cleft (arrow) is held closed with tweezers and the clamp. al, anterior leaflet; sl, septal leaflet.

During surgery, a cleft between the septal and anterior leaflets, in addition to the restriction of the septal leaflet, was discovered (*Figure 5*). The leaflets were not calcified. The patient underwent tricuspid valve reconstruction, the cleft was closed, and annuloplasty with 34-mm-ring implantation was performed.

Three years post-surgery, the patient is asymptomatic without palpitations or dyspnoea. Twenty-four hour Holter-monitoring shows AF with mean ventricular rate of 76 b.p.m. and no AFL. The current medication includes phenprocoumon, bisoprolol 2.5 mg/day, and ramipril 5 mg/day. Transthoracic echocardiography indicates dilated RA and RV with only mild TR.

## Discussion

This case highlights the importance of considering structural heart diseases in patients with recurrent AFL despite successful ablations and subsequent progression to AF. It also advocates for the use of 3D-TEE in evaluating right-heart-chamber enlargement of unknown aetiology.

Differential diagnoses for dilated RA and RV include ASD, Ebstein anomaly and arrhythmogenic right ventricular cardiomyopathy, all of which may be associated with AFL.^6,7^ These conditions were excluded by echocardiography and cMR. However, the assessment of the tricuspid valve remained incomplete. Due to the complex anatomy of the tricuspid valve, with its saddle-shaped annulus, visualization of all three leaflets in a single plane is challenging. 3D-TEE facilitates a simultaneous assessment of the leaflets, commissures, and valve coaptation in an en-face view.^3^ The absence of 3D-TEE in our hospital led to delays in identifying the aetiology of the TR and may have contributed to the incomplete postoperative recovery.

Cavotricuspid isthmus-ablation has an acute success-rate of 95% and a long-term success rate of 90%.^1^ These success-rates are comparable for AFL-ablation in patients with and without structural heart disease.^2,8^ However, the several attempts trying to treat the arrhythmia with ablations might have distracted us from recognizing the underlying problem and thus lead to delay in diagnosis and therapy.

In the literature, three cases are reported with AFL due to right-heart-chamber dilation. In one case, a left-to-right shunt from a pulmonary vein to the superior vena cava was corrected, leading to decreased heart size and restored sinus rhythm.^9^ Another case involved TR from traumatic chordal rupture, with the patient regaining sinus rhythm after tricuspid valve repair.^10^ The last patient with Ebstein anomaly, too fragile for surgery, was treated with CTI-ablation.^11^ In our patient, the cleft and restriction of the tricuspid leaflets are likely congenital or late consequences of the polytrauma.^12^ If we had come up with the cause of the right-heart-dilatation earlier, the patient might have retained sinus rhythm without ablations.

The pathophysiology linking AFL or AF to TR remains poorly understood. A rat model demonstrated that conditions affecting the right-heart-chambers create a substrate for AF maintenance by re-entrant activity in the RA with RA-fibrosis and conduction abnormalities.^13^ The relationship between AF and TR is bidirectional; TR and AF interact, sustaining each other through volume and pressure overload, tachycardiomyopathy and neurohumoral factors that remodel the myocardium and valve annulus. Both AF and AFL can act as markers and triggers for TR progression.^14^ In our patient, the worsening atrial stress caused by TR likely contributed to the progression of atrial arrhythmias despite previous ablations.

The ESC guidelines recommend surgical repair of severe TR as soon as RV-dilatation is observed.^15^ In this case, there was a 10-year delay between the detection of RV-dilatation and surgery (*Figure 1*). Post-operatively, RA and RV remained dilated, and AF persisted. It is likely that the remodelling of the right-heart-chambers had progressed too far to be reversible.

## Supplementary Material

ytae675_Supplementary_Data

## Data Availability

Data regarding the patient’s history and course are available in the records of Klinik Landstrasse and Klinik Floridsdorf, Vienna, and will be shared on reasonable request to the corresponding author.

## Lead author biography

Taemi Yoshida is a cardiologist specialized in electrophysiology and arrhythmia-therapy, working at Klinik Landstrasse, a public hospital located centrally in Vienna. Her aim in her carrier is to maintain a wide view in internal medicine and to take care of the patients in all aspects.

## Supplementary material

**Consent:** The authors confirm that written informed consent for submission and publication of this case report, including images and associated text, has been obtained from the patient.

**Funding:** None declared.
